# Elevated risk of thrombophilia in agenesis of the vena cava as a factor for deep vein thrombosis

**DOI:** 10.1186/s13023-014-0223-4

**Published:** 2015-01-21

**Authors:** Tolga Atilla Sagban, Rüdiger E Scharf, Markus U Wagenhäuser, Alexander Oberhuber, Hubert Schelzig, Klaus Grabitz, Mansur Duran

**Affiliations:** Department of Vascular and Endovascular Surgery, Heinrich Heine University Düsseldorf, Moorenstraße.5, 40225 Düsseldorf, Germany; Department for Hemostasis and Transfusion Medicine, Heinrich Heine University Düsseldorf, Moorenstraße.5, 40225 Düsseldorf, Germany

**Keywords:** Vena cava anomaly, Agenesis, Deep venous thrombosis, Thrombophilia

## Abstract

**Background:**

Congenital absence of the inferior vena cava (AIVC) is a rare malformation which may be associated with an increased risk for deep vein thrombosis (DVT). However, the role of thrombophilia in AIVC and DVT is unknown.

**Methods:**

Between 1982 and 2013 41 patients (12 female, 29 male, mean age 28 S.D. 11 years) were detected at the University of Düsseldorf, Germany, with AIVC. Based on medical history, clinical examination, imaging and coagulation studies, we performed on this collective a risk characterisation. Extensive literature research added further 123 published cases during 1993 and 2013. AIVC-patients were compared with iliocaval DVT-patients without AIVC (n = 168) treated during the same period in our clinic (90 female, 78 male, mean age 38 S.D. 17 years).

**Results:**

In contrast to classical DVT younger men were more often affected. Factor-V-Leiden-mutation, 5,10-methylenetetrahydrofolate reductase (MTHFR) polymorphism and hyperhomocysteinemia individually are associated with an increased risk of DVT in patients with AIVC. Aplasia/hypoplasia of the right or left kidney is also associated with IVCA.

**Conclusions:**

AIVC should be considered in young patients who present with DVT involving the vena cava. Analysis of publications with AIVC and our patients yielded a typical spectrum of AIVC-associated DVT characteristics: AIVC occurs in young male adults, is revealed by proximal DVT, not necessarily accused by precipitating factors like immobilisation, and is mostly located bilateral. Hereditary coagulation abnormalities seem to be more often a contributing factor for DVT in AIVC.

**Electronic supplementary material:**

The online version of this article (doi:10.1186/s13023-014-0223-4) contains supplementary material, which is available to authorized users.

## Background

The formation of the inferior vena cava (IVC) in the human embryo is a complex process with fusion and regression of three paired veins which develop around the sixth gestational week [1]. The posterior cardinal veins (PCV) drain the venous flow from the medial and caudal portions of the dorsal wall of the embryo into the venous sinus of the primitive heart through Cuvier’s veins. On the other hand, the omphalomesenteric veins, give rise to hepatic sinusoids, which lead directly to the coronary sinus via the hepatocardiac ducts. The primitive kidney or mesonephros is drained by the ipsilateral subcardinal veins (SV), which drain into the PCV. Between the two SV and the omphalomesenteric system numerous anastomoses exist, which give rise to the intrahepatic segment of the IVC. At this stage the involution of the PCV and SV begins, though the SV are only partially obliterated, maintaining their distal portion, which gives rise to the corresponding gonadal vein on the left and to the renal and postrenal segments of the IVC on the right. At the same time as the regression of the SV and PCV, a new system of SV develops designed to drain the dorsal wall of the embryo. These later veins develop later to the azygos and hemiazygos venous systems.

Malformations of the IVC are unusual and include an interruption of the IVC with azygos and hemiazygos vein continuation [2,3]. 90% of those cases involve suprarenal defects and only 6% involve the renal or infrarenal segments. Absence of the infrarenal segment of the IVC (AIVC) is an extremely rare anomaly [4]. The reasons for such a developmental failure are unclear. Most researchers believe that the cause lies in embryonic dysgenesis affecting separate segments or the entire IVC [5-7]. Others suggest that AIVC is not embryonic in origin, rather the result of intrauterine or perinatal thrombosis [8,9].

Until now only case reports or small collectives were reported for AIVC. Normally these malformations are well collateralized because of their presence since embryonic development. But some of them come to medical attention because of deep vein thrombosis (DVT). Although association with hyperhomcysteinemia [10] and factor-V-Leiden have been reported [11], in literature described collectives are very small and partly contrary [13]. Only limited data is available on the role of thrombophilia associated with AIVC [10-12]. To enlarge the small cohort of our AIVC patients (n = 41), we performed a Medline research reporting about cases with AIVC and could identify 124 additional AIVC patients with tested thrombophilia to ours (Additional file 1).

## Methods

This study was approved by the local ethics committee of the University Hospital of Düsseldorf, Germany and informed consent was obtained from every single patient treated and investigated in our department, which included permission to use data from chart review and genetic investigations for thrombophilc factors. The study was performed in accordance with the Helsinki Declaration.

### Subjects

We studied 209 consecutive patients between January 1982 and June 2013 with a history of ileo-femoral DVT with involvement of the vena cava. In this collective, 41 individuals with an AIVC were identified. Patients with ilio-femoral, vena cava involving DVT without AIVC (NoAIVC; n = 168) served as control group.

All patients were evaluated for determination of thrombophilic and other abnormalities. In both, the patients with a history of DVT and/or patients with an AIVC, blood samples were obtained after the diagnosis of DVT or/and AIVC.

We compared and added our results with those reported available on Medline database with sufficient relevant data. References were identified through search of the Medline database using the terms: “inferior vena cava”, “absence”, “anomaly”, “agenesis” and “atresia”. 68 publications could be identify, mostly case reports from 1993 until 2013, adding information of 124 patients with AIVC to our collective (Additional file 1). Publications without information about patients’ thrombophilia were disclosed. The references finally retained were chosen based on their relevance to topics addressed herein and to answer several questions important in clinical practice: demographic data of the AIVC population, clinical DVT presentation, imaging for AIVC diagnosis, additional anatomical anomalies, contribution of thrombophilia screening and therapeutic approach.

All patients treated in our clinic, had an objectively diagnosed episode of DVT diagnosed by Doppler ultrasonography, computed tomography or magnetic resonance imaging and/or by venography. These patients were treated with anticoagulation or when feasible by surgery in combination with anticoagulation [13].

### Laboratory tests

All patients in our clinic were screened for inherited and acquired defects in blood coagulation. We determined the activities of lupus anticoagulant and hyperhomocysteinemia in plasma, and performed a genetic analysis to determine factor-V-Leiden-gene-mutation, the G20210A prothrombin-gene-mutation, and the gene encoding C677T 5,10-methylenetetrahydrofolate reductase (MTHFR)-mutation. Lupus anticoagulant was measured with the DVV test and DVV confirm test (American Diagnostica, Greenwich, Conn.).

### Genetic analysis

DNA was extracted from peripheral-blood leukocytes according to standard protocols with the use of the Chelex system (Bio-Rad, Munich, Germany). The presence of factor-V-Leiden was determined by allele-specific restriction-enzyme analysis [14]. For confirmation of the genotypes, an oligonucleotide-ligation assay was performed [15]. The results of the oligonucleotide-ligation assay were in 100 percent concordance with the results of genotypic analysis, as determined by an allele specific restriction-enzyme assay. The G20210A prothrombin-gene-mutation was identified by allele-specific restriction-enzyme analysis [16]. Screening for the presence of the C677T MTHFR-polymorphism was performed by the method described by Frosst et al. [17].

### Statistical analysis

The SPSS statistical package (SPSS for Windows, version 19.0 SPSS Inc., Chicago, IL., USA) was used for all statistical analyses. Measure of variation is reported as standard deviation. Depending on the type of data, the Wilcoxon rank-sum test, chi-square analysis, or Fisher’s exact test (two-tailed) was used to assess the differences between the groups. Multivariate analyses, including the calculation of predictive values, were performed with the use of a stepwise logistic-regression procedure.

## Results

The characteristics of all patients are presented in Table 1 (details of patients derived by literature research is presented under Additional file 1).

**Table 1:** **Characteristics of the patients with an aivc in this paper and in literature, compared with noaivc**

**Characteristics**	**Patients with AIVC and DVT in this paper (n = 41)**	**Patients with AIVC in literature research (n = 124)**	**Patients with NoAIVC (n = 168)**	**P Value of AIVC in this paper and in literature (n = 165) vs. NoAIVC (n = 168)**
**Sex male/female (male in %)**	29/12 (70.7%)^†^	93/31 (75.0%)^†^	78/90 (46.4%)	<0.0001
**Mean age at appearance of**				
**DVT (years ± SD)**	28 ± 11.1^†^	28 ± 11.0^†^	38 ± 17.3	<0.0001
**Range (min., max.)**	16-58	8-67	8-84	
**preciptating factors for DVT -**				
**No. (%)**	25/41 (61.0%)^†^	52/122 (42.6%)^†^*	130/168 (77.4%)	<0.0001
**Left sided DVT (%)**	4/41 (9.8%)^†^	26/124 (21.0%)^†^	83/168 (49.4%)	<0.0001
**Right sided DVT (%)**	12/41 (29.3%)^†^	30/124 (24.2%)^†^	28/168 (16.7%)	0.049
**Both sided DVT (%)**	25/41 (61.0%)^†^	59/124 (47.6%)^†^	57/168 (33.9%)	0.002

**DVT** deep vein thrombosis. **AIVC** inferior vena cava agenesia. **NoAIVC** iliofemoral DVT with involvement of inferior vena cava without AIVC. **SD** standard deviance.

*in two cases not reported in literature.

^†^The difference between the AIVC groups was not significant.

### AIVC in our clinic and in literature

41 patients with AIVC could be identified at the University of Düsseldorf, Germany. Mean age was 28 ± 11.1 years (min. 16, max. 58 years; 29 male and 12 female). Medline database research revealed additional 124 AIVC patients reported in 68 articles. Mean age here was 28.0 ± 11.0 years (min. 8, max. 67 years; 93 male). Age difference and male dominance in both groups was not statistical significant. While in our department 36.6% of AIVC patients (15/41 patients) were surgical treated with a prosthetic bypass, anticoagulation and compressing stockings [13], in literature only three reported bypasses could be identified [9,18,19]. Thrombolysis was reported as treatment of first choice in 14 patients [20-28]. Three publications implied no information about their treatment, whereas the majority of the published cases were treated conservatively with anticoagulation and compressing stockings (n = 104/124).

DVT of the legs was present in all AIVC cases in our clinic. A left sided DVT was evaluable in 9.8% (4/41 patients), right sided in 29.3% (12/41 patients). Both sided affection was dominant with 61.0% in our AIVC patients (25/41 patients). There was a trend toward a higher prevalence of both sided DVT in our patients with AIVC than among the AIVC patients in literature (prevalence, 61.0% vs. 47.6%; p = 0.10) but reached no significance.

In the here employed literature, left sided DVT could be diagnosed in 21.0% (n = 26/124 patients), right sided in 30/124 patients (24.2%). Both sided affections of DVT was present in 59/124 patients (47.6%). Compared to our AIVC-group, DVT pattern was not significant different in literature.

Precipitating factors like immobilization, use of oral contraceptives, etc. were more often documented in NoAIVC (130/168) than in AIVC (77/163; 77.4% vs. 47.3%; p < 0.0001).

### NoAIVC

168 patients with NoAIVC served here as control group. Mean age was 38.0 ± 17.3 years (min. 8, max. 84 years). AIVC patients compared to NoAIVC patients were in average 10 years younger (p < 0.0001). Male dominance was also notable in AIVC (73.9% vs. 46.4%; p < 0.0001). In NoAIVC, right sided DVT was seen in 16.7% vs. 25.4% in AIVC, both sided in 33.9% vs. 50.9% in AIVC. Atypical site of DVT was significant for AIVC (right sided: p = 0.049; both sided: p = 0.002), whereas left sided DVT was more often observed in NoAIVC (49.4% vs. 18.3% in AIVC; p < 0.0001; Table 1).

### Other organ anomalies in AIVC and NoAIVC

In many reported cases, AIVC was associated with other anatomic disorders. The most recognized anomaly in AIVC was aplasia of the right kidney (6.0% vs. 0% in NoAIVC; p = 0.002), secondly, hypoplasia of the left kidney in 2.7% of AIVC patients (vs. 0% in NoAIVC; p = 0.042). Polysplenia was just as well seen in 2.0% of AIVC patients (vs. 0% in NoAIVC; p = 0.080; n.s.). May-Thurner syndrome was only observed in five NoAIVC-patients (3.0% vs. 0% in AIVC; p = 0.026). A preduodenal portal vein was only observed in two cases of AIVC and not statistical significant represented (p = 0.176; Table 2).

**Table 2:** **Prevalence of combined organ anomalies**

**Organ or system anomalies**	**Patients with AIVC in this paper (n = 41)**	**Patients with AIVC in literature (n = 108/124; 16/124 NR** ^**†**^ **)**	**Patients with NoAIVC (n = 168)**	**P Value of AIVC in this paper and in literature (n = 149) vs. NoAIVC (n = 168)**
**Hypoplasia/aplasia of left kidney (%)**	1/41 (2.4%)	3/108 (2.8%)	0/168 (0.0%)	0.042
**Hypoplasia/aplasia of right kidney (%)**	2/41 (4.9%)	7/108 (6.5%)	0/168 (0.0%)	0.002
**Polysplenia (%)**	1/41 (2.4%)	2/108 (1.9%)	0/168 (0.0%)	0.080
**Preduodenal portal vein (%)**	1/41 (2.4%)	1/108 (0.9%)^‡^	0/168 (0.0%)	0.176
**May-Thurner syndrome (%)**	0/41 (0.0%)	0/108 (0.0%)	5/168 (3.0%)	0.026

**AIVC** inferior vena cava agenesia. **NoAIVC** iliofemoral DVT with involvement of inferior vena cava without AIVC. **NR** not reported.

^†^In 16 of 124 reported cases in literature organ anomalies were not reported (NR); comparisons were calculated with 108 patients.

^‡^Absence of portal vein.

### Prevalence of inherited risk factors

There was no significant difference in the prevalence of factor-V-Leiden, prothrombin-gene-mutation, MTHFR-gene-mutation, and hyperhomocysteinemia between the 41 AIVC patients in our clinic and the 124 AIVC patients derived from literature research. Homozygoty for factor-V-Leiden or prothrombin-gene-mutation was not detected. In AIVC, factor-V-Leiden-mutation was heterozygous in 27/165 patients and in 8/168 in NoAIVC. Prothrombin-gene-mutation was heterozygous analysed in 9/165 AIVC and in 3/168 NoAIVC patients. Except of 2/168 NoAIVC individuals, all MTHFR-mutations were heterozygous (in AIVC 25/165; in NoAIVC 4/168). None of the patients with a history of AIVC had both, factor-V-Leiden and prothrombin-gene-mutation. A combination of two or more thrombophilic factors in-between the groups showed no significant difference (Table 3).

**Table 3:** **Prevalence of hereditary coagulation defects**

	**Patients with AIVC in this paper (n = 41)**	**Patients with AIVC in literature research (n = 124)**	**Patients with NoAIVC (n = 168)**	**P Value of AIVC in this paper and in literature (n = 165) vs. NoAIVC (n = 168)**
**Factor V Leiden (%)**	11/41 (26.8%)	16/124 (12.9%)	8/168 (4.8%)^†^	0.001
**Prothrombin gene mutation (%)**	1/41 (2.4%)	8/124 (6.5%)	2/168 (1.2%)	0.146
**MTHFR gene mutation (%)**	15/41 (36.6%)	6/124 (4.8%)	6/168 (3.6%)^‡^	0.005
**Homocysteinemia (%)**	15/41 (36.6%)	5/124 (4.0%)	5/168 (3.0%)	<0.0001
**Lupus anticoagulant (%)**	2/41 (4.9%)	4/124 (3.2%)	1/168 (0.6%)	0.053
**Two thrombophilic factors positive (%)**	9/41 (22.0%)	7/124 (5.6%)	4/168 (2.4%)	0.075
**Three and more thrombophilic factors positive (%)**	8/41 (19.5%)	1/124 (0.8%)	3/168 (1.8%)	0.086

**AIVC** inferior vena cava agenesia. **NoAIVC** iliofemoral DVT with involvement of inferior vena cava without AIVC.

^†^One case homozygous for factor-V-Leiden-gene-mutation. ^‡^Two cases homozygous for MTHFR-gene-mutation.

A strong statistic trend was present for lupus anticoagulant, detected in 6/165 AIVC patients vs. 1/168 in NoAIVC (3.6% vs. 0.6%; p = 0.053), but reached no significance (Table 3). Significant difference could be calculated for prevalence of factor-V-Leiden and hyperhomocysteinemia between AIVC and NoAIVC patients (prevalence of factor-V-Leiden, 16.4% vs. 4.8%; p = 0.001; prevalence of hyperhomocysteinemia, 12.1% vs. 3.0%; p < 0.0001; Tables 3 and 4). The prevalence of prothrombin-gene-mutation among the AIVC patients compared with NoAIVC patients was not significant (prevalence, 5.5% vs. 1.2%; p = 0.171; Table 4). By contrast, the MTHFR-gene-mutation was predominantly associated with AIVC (prevalence, 12.7% vs. 3.6% in NoAIVC patients; p = 0.005; Table 4).

**Table 4:** **Prevalence of hereditary coagulation defects in patients with aivc and in patients with noaivc**

**Characteristic**	**Prevalence among patients with NoAIVC (n = 168)**	Prevalence among patients with AIVC (n = 165)*	**P value** ^**†**^ **(NoAIVC vs. AIVC)**
	**Percent**		
**Gender (male)**	46.4	73.9	<0.0001
**Factor V Leiden**	4.8	16.4	0.001
**MTHFR gene mutation**	3.6	12.7	0.005
**Homocysteinemia**	3.0	12.1	<0.0001
**Prothrombin gene mutation**	1.2	5.5	0.171
**Lupus anticoagulant**	0.6	3.6	0.053

**AIVC** inferior vena cava agenesia. **NoAIVC** iliofemoral DVT with involvement of inferior vena cava without AIVC.

*AIVC in this paper and in literature (n = 165).

^†^P values are for the univariate comparison between patients with NoAIVC and those with AIVC.

In a logistic-regression analysis in which adjustments were made for age and gender (Table 5) and in which hyperhomocysteinemia, presence of factor-V-Leiden- polymorphism, the prothrombin-gene-mutation, lupus anticoagulant and MTHFR-gene-mutation were included in the same model, only the presence of factor-V-Leiden-gene-mutation, hyperhomocysteinemia and MTHFR-gene-mutation could be identified as independent risk factors (Table 5). Localisation of DVT was also analysed. There was a statistical difference with regard to the prevalence or relative risk of both or right sided DVT between AIVC and NoAIVC patients (p = 0.049 resp. p = 0.002). Nevertheless, left sided DVT was predominantly associated with NoAIVC than with AIVC (prevalence, 18.2% among AIVC vs. 49.4% in NoAIVC; p < 0.0001). Aplasia of the right and hypoplasia of the left kidney were statistical significant in multivariate analyses for AIVC (Table 5).

**Table 5:** **Relative risk of aivc associated with hereditary coagulation defects and anatomic malformations**

**Risk factors in AIVC**	**Univariate analysis**		**Multivariate analysis** ^**†**^	
	**Relative risk (95% CI)**	**P value**	**Relative risk (95% CI)**	**P value**
**Age (years)**	27.98 (35.65-41.17)	<0.0001**	22.31 (32.62-39.89)	<0.0001
**Gender (male)**	3.274 (2.064-5.192)	<0.0001*	5.789 (3.131-10.701)	<0.0001
**Factor V Leiden**	3.913 (1.721-8.895)	0.001*	4.890 (1.647-7.517)	<0.0001
**MTHFR gene mutation**	3.354 (1.385-8.123)	0.005*	2.132 (1.016-7.072)	0.005
**Homocysteinemia**	6.641 (2.243-19.659)	<0.0001*	6.916 (2.544-19.814)	<0.0001
**Aplasia of right kidney**	58.4^††^	0.002*	64.8^††^	0.002
**Hypoplasia of left kidney**	36.1^††^	0.059*	46.7^††^	0.042
**Right sided DVT**	1.707 (0.999-2.918)	0.049*	1.663 (1.025-3.267)	0.049
**Both sided DVT**	2.019 (1.298-3.141)	0.002*	2.003 (1.305-3.349)	0.002

**DVT** deep vein thrombosis. **AIVC** inferior vena cava agenesia. **MTHFR** 5,10-methylenetetrahydrofolate reductase.

*Univariate analyses were performed with the use of the chi-square test. Multivariate analyses were performed with the use of the stepwise logistic-regression procedure. CI denotes confidence interval.

**Univariate analyses were performed with the use of the T-test. Multivariate analyses were performed with the use of the stepwise logistic-regression procedure. CI denotes confidence interval.

^†^All stepwise logistic-regression analyses were performed with the following variables: presence of factor-V-Leiden; presence of prothrombin-gene-mutation; presence of the MTHFR-gene-mutation; hyperhomocysteinemia; presence of Lupus anticoagulant; aplasia of the right kidney; hypoplasia of the left kidney; polysplenia; right sided DVT; both sided DVT; age; gender; the P values indicate the significance of each risk factor independently.

^††^Because no patient with NoAIVC had this combined defect, the estimated relative risk was calculated on the basis of the probability of a combined defect in these patients. For this reason, no confidence interval is given.

## Discussion

The factors that predispose to venous thrombosis were initially described by Virchow in 1856 [29]. Individuals with DVT usually have a predisposing risk factor, such as a genetic abnormality causing hypercoagulability, pregnancy, etc. AIVC should also be considered as a potential associated anatomic abnormality, lacking of a main venous conduit, which influences blood flow in the venous system [11]. Our results showed that precipitating factors like immobilization were less often an activator for DVT in AIVC patients. Younger male with atypical DVT (right or both sided DVT) are more often affected by AIVC presenting with signs and symptoms of an acute DVT without previous evidence of risk factors. Thrombophilia plays also an important role in AIVC patients although other authors published contrary findings based on case reports or small groups [11,12]. While factor-V-Leiden-gene-mutation can be found in a normal population roughly in 5%, a similar prevalence could be shown in our control group (NoAIVC patients 4.8%). In AIVC the prevalence is more than three times higher (16.4%). In our opinion this is not debted to a selection towards AIVC with DVT, because the here presented prevalence of factor-V-Leiden-mutation in NoAIVC is similar to the normal population. In addition, homozygosis for a common cytosine-to-thymidine mutation in the gene encoding MTHFR, C677T, is associated with high plasma homocysteine concentrations and venous thrombosis. There are conflicting results regarding the role of a homozygous 677TT MTHFR genotype as a risk factor for venous thromboembolism. Homozygosis for this polymorphism was associated with high plasma homocysteine concentrations and venous thrombosis in some studies [17,30,31] but not in others [32]. In our study, the 677TT MTHFR-genotype (homozygote) was only present in two cases of NoAIVC. All other MTHFR-positive testing’s were heterozygous with the 677CT MTHFR genotype combined with hyperhomocysteinemia. In our opinion this genotype in combination with metabolic anomaly has an influence on the risk of DVT; the prevalence of the 677CT MTHFR-genotype in AIVC was higher than in the group of NoAIVC. Like factor-V-Leiden, MTHFR-gene-mutation was predominant in AIVC, mostly in combination with hyperhomocysteinemia. The G20210A prothrombin-gene- mutation or the presence of lupus anticoagulant seems to play no role here.

We confirmed the importance of factor-V-Leiden as a risk factor for venous thromboembolism in AIVC. In addition, we found that factor-V-Leiden is a risk factor that is independent of other known determinants of thrombosis; specifically, it is independent of the G20210A prothrombin or MTHFR-gene-mutation, whereas MTHFR-mutation in combination with hyperhomocysteinemia is independently from factor-V-Leiden-mutation also a risk factor for DVT in AIVC.

The developmental cause of AIVC is debated: it’s maybe caused by embryonic dysgenesis or by an intrauterine insult during the perinatal period [6,33]. AIVC have been associated with other congenital anomalies, like renal hypoplasia/agenesis. This anatomic anomaly for the right kidney was found in our research in 6% of AIVC, but not in NoAIVC patients. The right renal hypoplasia is suggestive of a defect in the formation of the IVC in these segments, since the embryologic right SV does not drain the mesonephros [34]. A hypoplastic left kidney was also found here in AIVC (2.7%). However, it is not clear whether this represents a true congenital malformation or atrophy of a previously normal kidney due to long-standing poor perfusion or thrombosis secondary to vascular malformation. The question of hereditary is not clearly answerable, but two cases in our collective direct to this hypothesis. In one case, the son of a patient with AIVC was diagnosed with DVT bilateral. Computed tomography showed here also an AIVC in the same segment like his father. The second remarkable case was the asymptomatic twin-brother of an AIVC patient, who received imaging for other reasons. His AIVC pattern was identical to his twin brother. The thrombophilic patterns were identical in-between the relatives. Of course, these relatives with AIVC cannot be representative, but both give rise to a hereditary hypothesis of AIVC. Interestingly, factor-V-deficiency is caused by mutations in the F5 gene, located on the long (q) arm of chromosome 1 at position 23, nearby LEFTY2 (Left-Right Determination Factor 2), playing a role in left-right asymmetry determination of organ systems during development at cytogenetic location 1q42.1 [35]. Renal dysgenesis and MTHFR-mutation is also located on this chromosome [35]. Further genetic investigations could here emblaze the hereditary genesis of AIVC.

## Conclusions

This study shows that AIVC is associated with a higher prevalence of thrombophilia even compared to patients with iliofemoral DVT with involvement of inferior vena cava but without AIVC. AIVC should be considered in young patients who present with DVT involving the vena cava. Analysis of publications with AIVC and our patients yielded a typical spectrum of AIVC-associated DVT characteristics: AIVC occurs in young male adults, is revealed by proximal DVT, not necessarily accused by precipitating factors like immobilisation, and is mostly located bilateral. Hereditary coagulation abnormalities seem to be more often a contributing factor for DVT in AIVC.

## Additional file

Additional file 1:
**AIVC reported in literature and employed here in this study.**

## Abbreviations

AIVC: Inferior vena cava agenesis
DVT: Deep vein thrombosis
IVC: Inferior vena cava
MTHFR: 5,10-methylenetetrahydrofolate reductase
NoAIVC: Iliofemoral DVT with involvement of inferior vena cava without AIVC
PCV: Posterior cardinal veins
SV: Subcardinal veins
