# Supplementary material for: Elevated risk of thrombophilia in agenesis of the vena cava as a factor for deep vein thrombosis
Source: Orphanet J Rare Dis. 2015 Jan 21;10:3. doi: 10.1186/s13023-014-0223-4 (PMC4308084; doi:10.1186/s13023-014-0223-4)
Supplement: Additional file 1: — AIVC reported in literature and employed here in this study. [file 13023_2014_223_MOESM1_ESM.doc]

**Supplement data I: AIVC reported in literature and employed here in this study**

**DVT** deep vein thrombosis **NR** Not reported **Ø** no anomalies

| **Reference** | **Ref. Year** | **Age (years)** | **Gender** | **Combined anomalies** | **Site of DVT** | **Underlying coagulopathy** | **Precipitating factors** | **Treatment** |
| --- | --- | --- | --- | --- | --- | --- | --- | --- |
| Halbmeyer et al. [1] | 1993 | 24 | male | Ø | both legs | factor XII deficiency | NR | Anticoagulation |
| Saito et al. [2] | 1995 | 19 | male | Ø | left leg | no | yes | Anticoagulation |
| Dougherty et al. [3] | 1996 | 41 | male | Ø | right leg | no | no | Bypass surgery |
| Shah et al. [4] | 1996 | 30 | male | Ø | both legs | no | no | Anticoagulation |
| Salgado Ordonez et al. [5] | 1998 | 49 | male | aplasia/hypoplasia of right kidney | both legs | no | NR | Anticoagulation |
| Awartani et al. [6] | 1999 | 23 | female | Ø | NR | no | yes | Anticoagulation |
| Klessen et al. [7] | 1999 | 29 | male | Ø | right leg | no | yes | Anticoagulation |
| Timmers et al. [8] | 1999 | 37 | male | aplasia/hypoplasia of right kidney | right leg | no | no | Anticoagulation |
| Tiesenhausen et al. [9] | 1999 | 25 | male | Ø | right leg | no | yes | Anticoagulation |
|  |  | 20 | female | Ø | left leg | protein S deficiency | yes | Anticoagulation |
| Hamoud et al. [10] | 2000 | 30 | male | Ø | both legs | no | no | Anticoagulation |
| Slosman et al. [11] | 2001 | 47 | male | Ø | NR | no | no | NR |
| Körber et al. [12] | 2001 | 19 | male | Ø | both legs | no | no | Thrombolysis |
| Granel et al. [13] | 2001 | 29 | male | Ø | both legs | no | yes | Anticoagulation |
| Chee et al. [14] | 2001 | 23 | male | Ø | NR | no | no | Anticoagulation |
|  |  | 18 | female | Ø | both legs | heterozygous factor V Leiden  gene mutation | yes | Anticoagulation |
|  |  | 40 | male | Ø | both legs | no | no | Anticoagulation |
|  |  | 26 | female | Ø | both legs | no | no | Anticoagulation |
| Ramanathan et al. [15] | 2001 | 12 | female | Ø | left leg | no | no | Anticoagulation |
| Ruggeri et al. [16] | 2001 | 20 | male | Ø | right leg | no | no | Anticoagulation |
|  |  | 22 | male | Ø | both legs | no | no | Anticoagulation |
|  |  | 15 | male | Ø | right leg | no | no | Anticoagulation |
|  |  | 19 | female | Ø | both legs | no | no | Anticoagulation |
| Siragusa et al. [17] | 2001 | 28 | male | Ø | left leg | heterozygous factor V Leiden  gene mutation | no | Anticoagulation |
|  |  | 24 | female | Ø | both legs | heterozygous factor V Leiden  gene mutation | no | Anticoagulation |
| Tsuji et al. [18] | 2001 | 21 | male | aplasia/hypoplasia of right kidney | both legs | no | no | Thrombolysis |
| Obernosterer et al. [19] | 2002 | 25 | male | Ø | both legs | no | no | Anticoagulation |
|  |  | 24 | male | Ø | both legs | no | yes | Anticoagulation |
|  |  | 22 | female | Ø | left leg | protein S deficiency | yes | Anticoagulation |
|  |  | 20 | female | Ø | left leg | no | yes | Anticoagulation |
|  |  | 35 | male | Ø | both legs | no | no | Anticoagulation |
| Schneider et al. [20] | 2002 | 44 | male | Ø | left leg | heterozygous factor V Leiden  gene mutation | no | Anticoagulation |
| Parma et al. [21] | 2003 | 18 | male | Ø | both legs | heterozygous factor V Leiden  gene mutation, homozygous  MTHFR gene mutation | no | Anticoagulation |
| Sandercoe et al. [22] | 2003 | 20 | male | Ø | both legs | no | no | Anticoagulation |
| D'Aloia et al. [23] | 2003 | 40 | female | Ø | right leg | no | yes | Anticoagulation |
| Gayer et al. [24] | 2003 | 21 | male | Ø | both legs | heterozygous factor V Leiden  gene mutation, Lupus anticoagulant | no | Anticoagulation |
|  |  | 25 | male | Ø | both legs | heterozygous MTHFR gene mutation, hyperhomocysteinemia | no | Anticoagulation |
|  |  | 57 | male | Ø | left leg | hyperhomozysteinemia | no | Anticoagulation |
|  |  | 46 | male | aplasia/hypoplasia of right kidney | left leg | no | no | Anticoagulation |
|  |  | 25 | male | Ø | both legs | prothrombin gene mutation | no | Anticoagulation |
|  |  | 25 | male | Ø | both legs | prothrombin gene mutation | no | Anticoagulation |
|  |  | 25 | male | Ø | both legs | prothrombin gene mutation | no | Anticoagulation |
|  |  | 25 | male | Ø | both legs | no | yes | Anticoagulation |
|  |  | 19 | female | Ø | left leg | heterozygous factor V Leiden  gene mutation | yes | Anticoagulation |
| Motwani et al. [25] | 2004 | 19 | male | Ø | both legs | no | no | Anticoagulation |
| Cho et al. [26] | 2004 | 32 | male | Ø | right leg | no | no | Anticoagulation |
| Yun et al. [27] | 2004 | 39 | female | Ø | both legs | heterozygous MTHFR gene mutation, hyperhomocysteinemia | no | Anticoagulation |
| Lane [28] | 2005 | 38 | male | Ø | left leg | no | no | Anticoagulation |
| Sakellaris et al. [29] | 2005 | 10 | male | Ø | both legs | no | no | Anticoagulation |
| Takehara et al. [30] | 2005 | 67 | female | Ø | both legs | no | no | Anticoagulation |
| Koc et al. [31] | 2006 | 30 | male | Ø | both legs | heterozygous factor V Leiden  gene mutation | yes | Anticoagulation |
|  |  | 48 | male | Ø | right leg | no | yes | Anticoagulation |
|  |  | 14 | female | Ø | right leg | protein S deficiency, AT III deficiency | yes | Anticoagulation |
|  |  | 34 | male | polysplenia | right leg | no | yes | Anticoagulation |
|  |  | 24 | female | Ø | left leg | no | yes | Anticoagulation |
|  |  | 35 | female | Ø | right leg | no | yes | Anticoagulation |
|  |  | 53 | female | aplasia/hypoplasia of right kidney | both legs | no | yes | Anticoagulation |
|  |  | 21 | male | Ø | both legs | no | yes | Anticoagulation |
| Dean et al. [32] | 2006 | 16 | male | Ø | right leg | no | yes | Thrombolysis |
|  |  | 18 | female | Ø | right leg | no | yes | Thrombolysis |
| Simon et al. [33] | 2006 | 39 | male | Ø | left leg | no | no | Anticoagulation |
| Stratopoulos et al. [34] | 2006 | 21 | male | Ø | left leg | no | no | NR |
| Garcia-Fuster et al. [35] | 2006 | 20 | female | Ø | both legs | Lupus anticoagulant | yes | Anticoagulation |
|  |  | 27 | male | Ø | both legs | prothrombin gene mutation | yes | Anticoagulation |
|  |  | 27 | male | Ø | both legs | Lupus anticoagulant | yes | Anticoagulation |
|  |  | 27 | male | Ø | both legs | no | yes | Anticoagulation |
| Gil et al. [36] | 2006 | 14 | male | Ø | left leg | no | no | Anticoagulation |
| Ilijic et al. [37] | 2007 | 23 | female | Ø | right leg | no | yes | Anticoagulation |
| Dudeck et al. [38] | 2007 | 26 | male | Ø | both legs | no | yes | Anticoagulation |
| Evanchuk et al. [39] | 2008 | 26 | male | aplasia/hypoplasia of right kidney | left leg | no | yes | Anticoagulation |
| Vucicevic et al. [40] | 2008 | 22 | male | Ø | right leg | heterozygous MTHFR gene mutation, homozygous PAI-1  gene mutation | yes | Anticoagulation |
| Iqbal et al. [41] | 2008 | 54 | male | hypoplasia of left kidney | right leg | no | no | Anticoagulation |
| Clayburgh et al. [42] | 2008 | 50 | male | hypoplasia of left kidney | left leg | no | yes | Anticoagulation |
| Tofigh et al. [43] | 2008 | 38 | male | Ø | both legs | no | no | Bypass surgery |
| Waseem et al. [44] | 2008 | 14 | male | hypoplasia of left kidney | right leg | no | no | Anticoagulation |
| Sanchez Fernandez et al. [45] | 2008 | 17 | male | Ø | left leg | heterozygous factor V Leiden  gene mutation | no | Thrombolysis |
| Suh et al. [46] | 2008 | 62 | male | Ø | right leg | no | no | Anticoagulation |
| Kara et al. [47] | 2008 | 18 | male | Ø | right leg | no | yes | Anticoagulation |
| Kelly et al. [48] | 2009 | 24 | male | Ø | both legs | no | no | Anticoagulation |
| Vasco et al. [49] | 2009 | 36 | female | Ø | both legs | heterozygous factor V Leiden  gene mutation | no | Anticoagulation |
| Kondo et al. [50] | 2009 | 27 | male | Ø | both legs | no | no | Anticoagulation |
| Rose et al. [51] | 2009 | 33 | male | Ø | right leg | heterozygous factor V Leiden  gene mutation, heterozygous MTHFR gene mutation | yes | Anticoagulation |
|  |  | 34 | female | Ø | right leg | prothrombin gene mutation | yes | Thrombolysis |
| Schierl et al. [52] | 2009 | 28 | male | polysplenia | left leg | no | no | Anticoagulation |
| Guanella et al. [53] | 2009 | 30 | male | Ø | right leg | no | no | Anticoagulation |
|  |  | 15 | male | Ø | both legs | prothrombin gene mutation | no | Anticoagulation |
|  |  | 24 | male | Ø | both legs | no | no | Anticoagulation |
|  |  | 27 | male | Ø | right leg | heterozygous factor V Leiden  gene mutation | no | Anticoagulation |
|  |  | 12 | male | Ø | both legs | protein S deficiency | no | Anticoagulation |
|  |  | 17 | female | Ø | both legs | Lupus anticoagulant | yes | Anticoagulation |
|  |  | 15 | male | Ø | left leg | heterozygous factor V Leiden  gene mutation | no | Anticoagulation |
|  |  | 42 | male | Ø | both legs | no | no | Anticoagulation |
|  |  | 45 | male | Ø | both legs | no | no | Anticoagulation |
|  |  | 24 | male | Ø | right leg | no | no | Anticoagulation |
| Nichols et al. [54] | 2010 | 18 | female | Ø | left leg | no | yes | Anticoagulation |
| Ismail et al. [55] | 2010 | 8 | male | Ø | right leg | no | no | Anticoagulation |
| Singh et al. [56] | 2010 | 28 | male | Ø | left leg | factor VIII deficiency | no | Thrombolysis |
| Lambert et al. [57] | 2010 | 16 | male | NR | both legs | heterozygous factor V Leiden  gene mutation | yes | Anticoagulation |
|  |  | 26 | male | NR | both legs | prothrombin gene mutation | yes | Anticoagulation |
|  |  | 26 | male | NR | both legs | no | yes | Anticoagulation |
|  |  | 26 | male | NR | both legs | no | yes | Anticoagulation |
|  |  | 26 | male | NR | both legs | no | yes | Anticoagulation |
|  |  | 26 | male | NR | both legs | no | yes | Anticoagulation |
|  |  | 26 | male | NR | NR | no | yes | Anticoagulation |
|  |  | 26 | male | NR | NR | no | yes | Anticoagulation |
|  |  | 26 | female | NR | NR | no | no | Anticoagulation |
|  |  | 30 | female | NR | NR | no | no | Anticoagulation |
| O'Connor et al. [58] | 2011 | 20 | male | Ø | left leg | no | no | Anticoagulation |
| La Spada et al. [59] | 2011 | 35 | male | Ø | both legs | heterozygous MTHFR gene mutation, hyperhomocysteinemia | no | Bypass surgery |
| Nseir et al. [60] | 2011 | 33 | male | Ø | right leg | no | no | Anticoagulation |
| Garg et al. [61] | 2011 | 25 | male | aplasia/hypoplasia of right kidney | both legs | heterozygous factor V Leiden  gene mutation | no | Thrombolysis |
| Staubach et al. [62] | 2011 | 45 | male | Ø | both legs | prothrombin gene mutation | no | Anticoagulation |
| Sarlon et al. [63] | 2011 | 39 | male | Ø | both legs | no | no | Anticoagulation |
| Ganguli et al. [64] | 2011 | 18 | female | NR | both legs | AT III deficiency | yes | Thrombolysis |
|  |  | 15 | female | NR | right leg | no | yes | Thrombolysis |
|  |  | 21 | male | NR | both legs | no | no | Thrombolysis |
|  |  | 21 | male | NR | both legs | no | yes | Thrombolysis |
|  |  | 30 | female | NR | left leg | heterozygous factor V Leiden  gene mutation | yes | Thrombolysis |
|  |  | 22 | male | NR | left leg | no | yes | Thrombolysis |
| Zinser et al. [65] | 2012 | 50 | female | Ø | NR | no | yes | NR |
| Lavens et al. [66] | 2013 | 33 | female | Ø | right leg | no | yes | Anticoagulation |
| Skeik et al. [67] | 2013 | 23 | male | Ø | NR | no | no | Anticoagulation |
| Yugueros et al. [68] | 2013 | 32 | male | Ø | right leg | no | no | Anticoagulation |
|  |  | 30 | male | Ø | left leg | no | no | Anticoagulation |
| **Patients treated with AIVC in Düsseldorf** | 2014 | 23 | male | Ø | right leg | heterozygous factor V Leiden gene mutation, | no | Anticoagulation |
|  |  | 58 | female | aplasia/hypoplasia of right kidney, polysplenia | left leg | heterozygous factor V Leiden gene mutation, heterozygous MTHFR gene mutation, hyperhomocysteinemia | yes | Anticoagulation |
|  |  | 47 | male | Ø | right leg | heterozygous MTHFR gene mutation, hyperhomocysteinemia | yes | Anticoagulation |
|  |  | 27 | male | Ø | both legs | no | yes | Bypass |
|  |  | 23 | male | Ø | both legs | homozygous MTHFR gene mutation, hyperhomocysteinemia | yes | Bypass |
|  |  | 25 | male | Ø | left leg | heterozygous factor V Leiden gene mutation, heterozygous MTHFR gene mutation, hyperhomocysteinemia | yes | Anticoagulation |
|  |  | 16 | male | Ø | both legs | no | yes | Anticoagulation |
|  |  | 17 | male | Ø | both legs | no | no | Anticoagulation |
|  |  | 30 | male | Ø | right leg | homozygous MTHFR gene mutation, hyperhomocysteinemia | no | Anticoagulation |
|  |  | 57 | male | Ø | right leg | no | yes | Anticoagulation |
|  |  | 22 | male | Ø | right leg | no | no | Bypass |
|  |  | 21 | female | Ø | both legs | no | no | Bypass |
|  |  | 17 | male | Ø | both legs | heterozygous MTHFR gene mutation, hyperhomocysteinemia | no | Bypass |
|  |  | 48 | male | Ø | both legs | heterozygous factor V Leiden gene mutation, heterozygous MTHFR gene mutation, hyperhomocysteinemia | yes | Bypass |
|  |  | 39 | female | Ø | both legs | no | yes | Bypass |
|  |  | 37 | male | Ø | right leg | heterozygous MTHFR gene mutation, hyperhomocysteinemia | no | Bypass |
|  |  | 32 | male | Ø | both legs | heterozygous MTHFR gene mutation, hyperhomocysteinemia | no | Bypass |
|  |  | 20 | male | Ø | both legs | no | no | Bypass |
|  |  | 29 | male | Ø | both legs | no | no | Bypass |
|  |  | 20 | male | Ø | both legs | prothrombin gene mutation | yes | Bypass |
|  |  | 17 | female | Ø | both legs | no | yes | Bypass |
|  |  | 22 | female | Ø | right leg | heterozygous factor V Leiden gene mutation, heterozygous MTHFR gene mutation, hyperhomocysteinemia | yes | Bypass |
|  |  | 28 | female | Ø | both legs | heterozygous factor V Leiden gene mutation, heterozygous MTHFR gene mutation, hyperhomocysteinemia | yes | Bypass |
|  |  | 24 | female | Ø | left leg | heterozygous factor V Leiden gene mutation, heterozygous MTHFR gene mutation, hyperhomocysteinemia | yes | Bypass |
|  |  | 43 | male | Ø | both legs | no | yes | Bypass |
|  |  | 18 | female | Ø | both legs | no | yes | Anticoagulation |
|  |  | 22 | female | Ø | both legs | heterozygous MTHFR gene mutation, hyperhomocysteinemia | yes | Anticoagulation |
|  |  | 28 | male | hypoplasia of left kidney | right leg | no | no | Bypass |
|  |  | 29 | male | Ø | both legs | Lupus anticoagulant | yes | Bypass |
|  |  | 39 | male | Ø | both legs | Lupus anticoagulant | no | Bypass |
|  |  | 22 | male | Ø | right leg | heterozygous factor V Leiden gene mutation | yes | Bypass |
|  |  | 23 | male | Ø | right leg | heterozygous MTHFR gene mutation, hyperhomocysteinemia | no | Bypass |
|  |  | 22 | male | Ø | both legs | no | no | Bypass |
|  |  | 16 | male | Ø | both legs | no | no | Bypass |
|  |  | 23 | female | Ø | both legs | no | yes | Thrombolysis |
|  |  | 21 | male | Ø | right leg | heterozygous MTHFR gene mutation, hyperhomocysteinemia | yes | Anticoagulation |
|  |  | 25 | female | Ø | left leg | heterozygous factor V Leiden gene mutation | yes | Anticoagulation |
|  |  | 18 | male | Ø | both legs | no | yes | Thrombectomy |
|  |  | 26 | male | Ø | both legs | no | no | Anticoagulation |
|  |  | 32 | female | aplasia/hypoplasia of right kidney | both legs | heterozygous factor V Leiden gene mutation | yes | Anticoagulation |
|  |  | 48 | male | Ø | right leg | heterozygous factor V Leiden gene mutation | yes | Anticoagulation |

**References of AIVC reported in literature and employed here in this study:**

1. Halbmayer WM, Radek J, Duschet P, et al. Rezidivierende Venenthrombosen bei Vena-cava-inferior-Hypoplasie und Faktor-XII-Mangel. DMW 1993; 118: 1561-1566.
2. Saito H, Sano N, Kaneda I, et al. Multisegmental anomaly of the inferior vena cava with thrombosis of the left inferior vena cava. Cardiovasc Intervent Radiol 1995; 18: 410-413.
3. Dougherty MJ, Calligaro KD, DeLaurentis DA. Congenitally absent inferior vena cava presenting in adulthood with venous stasis and ulceration: A surgically treated case. J Vasc Surg 1996; 23: 141-146.
4. Shah NL, Shanley CJ, Prince MR, et al. Deep venous thrombosis complicating a congenital absence of the inferior vena cava. Surgery 1996; 120: 891-896.
5. Salgado-Ordonez F, Gavilan Carrasco JC, Bermudez Recio FJ, et al. Absence of the inferior vena cava causing repeated deep venous thrombosis in an adult: A case report. Angiology 1998; 49: 951-956.
6. Awartani KA, McComb PF. Ovarian cyst formation and congenital absence of the inferior vena cava: case report. Clin Exp Obstet Gynecol 1999; 26: 147-148.
7. Klessen C, Deutsch HJ, Karasch T, et al. Thrombose der tiefen Bein- und Beckenvenen bei angeborener Agenesie der Vena cava inferior. DMW 1999; 124: 523-526.
8. Timmers GJ, Falke TH, Rauwerda JA, et al. Deep vein thrombosis as apresenting symptom of congenital interruption of the inferior vena cava. Int J Clin Pract 1999; 53: 75-76.
9. Tiesenhausen K, Amann W, Thalhammer M, et al. Aplasia of the vena cava inferior as cause for recurring thrombosis of the lower extremities and pelvic veins. VASA 1999; 28: 289-292.
10. Hamoud S, Nitecky S, Engel A, et al. Hypoplasia of the inferior vena cava with azygous continuation presenting as recurrent leg deep vein thrombosis. Am J Med Sci 2000; 319: 414-416.
11. Slosman F, Schmid MR, Pfammatter T. Agenesis of the hepatic segment of the inferior vena cava with portal continuation. AJR [Am J Roentgenol](http://www.ncbi.nlm.nih.gov/pubmed/?term=Slosman+F%2C+Schmid+MR%2C+Pfammatter+T) 2001; 177: 120-122.
12. Körber T, Petzsch M, Placke J, et al. Akute Becken-Beinvenenthrombose bei Agenesie des renalen Segments der Vena cava inferior. Z Kardiol 2001 90: 52-57.
13. Granel B, Serratrice J, Bartoli JM, et al. Bilateral iliac thrombosis affter seat belt-related trauma revealing hypoplasia of the inferior vena cava—a case report. Angiology 2001; 53: 3593-62.
14. Chee YL, Culligan DJ, Watson HG. Inferior vena cava malformation as a risk factor for deep venous thrombosis in the young. Brit J Haematol 2001; 114: 878-880.
15. Ramanathan T, Hughes TMD, Richardson AJ. Perinatal inferior vena cava thrombosis and absence of the inferior vena cava. J Vasc Surg 2001; 33: 1097-1099.
16. Ruggeri M, Tosetto A, Castaman G, et al. Congenital absence of the inferior vena cava: a rare risk factor for idiopathic deep-vein thrombosis. Lancet 2001; 357: 441.
17. Siragusa S, Anastasio R, Falaschi F et al. Congenital absence of inferior vena cava. Lancet 2001; 357: 1711.
18. Tsuji Y, Inoue T, Murakami H, et al. Deep vein thrombosis caused by congenital interruption of the inferior vena cava: A case report. Angiology 2001; 52: 721-725.
19. Obernosterer A, Aschauer M, Schnedl W, et al. Anomalies of the inferior vena cava in patients with iliac venous thrombosis. Ann Intern Med 2002; 136: 37-41.
20. Schneider JG, Eynatten MV, Dugi KA, et al. Recurrent deep venous thrombosis caused by congenital interruption of the inferior vena cava and heterozygous factor V Leiden mutation. J Intern Med 2002; 252: 276-280.
21. Parma M, Belotti D, Marinoni S, et al. Congenital absence of the inferior vena cava and genetic coagulation abnormalities: a rare associated risk factor for recurrent idiopathic deep vein thrombosis. Clin Appl Thromb Hemost 2003; 9: 347-348.
22. Sandercoe GD, Brooke-Cowden GL. Developmental anomaly of the inferior vena cava. ANZ J Surg 2003; 73: 356-360.
23. D’Aloia A, Faggiano P, Fiorina C, et al. Absence of inferior vena cava as arare cause of deep vein thrombosis complicated by liver and lung embolism. Int J Cardiol 2003; 88: 327-329.
24. Gayer G, Luboshitz J, Hertz M, et al. Congenital anomalies of the inferior vena cava revealed on CT in patients with deep vein thrombosis. AJR [Am J Roentgenol](http://www.ncbi.nlm.nih.gov/pubmed/?term=Slosman+F%2C+Schmid+MR%2C+Pfammatter+T) 2003; 180: 729-732.
25. Motwani J, Rose PE, Shatwell W. An unusual case of venous thromboembolism. Br J Haematol 2005; 128: 1.
26. Cho BC, Choi HJ, Kang SM, et al. [Congenital absence of inferior vena cava as a rare cause of pulmonary thromboembolism.](http://www.ncbi.nlm.nih.gov/pubmed/15515211) Yonsei Med J 2004; 45: 947-951.
27. Yun SS, Kim JI, Kim KH, et al. Deep venous thrombosis caused by congenital absence of inferior vena cava, combined with hyperhomocysteinemia. Ann Vasc Surg 2004; 18: 124-129.
28. Lane DA. Congenital hypoplasia of the inferior vena cava: an underappreciated cause of deep venous thromboses among young adults. Mil Med 2005; 170: 739-742.
29. Sakellaris G, Tilemis S, Papakonstantinou O, et al. Deep venous thrombosis in a child associated with an abnormal inferior vena cava. Acta Paediatr 2005; 94: 242-244.
30. Takehara N, Hasebe N, Enomoto S, et al. Multiple and recurrent systemic thrombotic events associated with congenital anomaly of inferior vena cava. J ThrombThrombolysis 2005; 19: 101-103.
31. Koc Z, Oguzkurt L. Interruption or congenital stenosis of the inferior vena cava: prevalence, imaging, and clinical findings. Eur J Radiol 2007; 62: 257-266.
32. Dean SM, Tytle TL. Acute right lower extremity iliofemoral deep venous thrombosis secondary to an anomalous inferior vena cava: a report of two cases. Vasc Med 2006; 11: 165-169.
33. Simon RW, Amann-Vesti BR, Pfammatter T, et al. Congenital absence of the inferior vena cava: a rare risk factor for idiopathic deep-vein thrombosis. J Vasc Surg 2006; 44: 416.
34. Stratopoulos C, Pitsios C, Valenti P, et al. Deep vein thrombosis due to absence of inferior vena cava. NZMJ 2006; 119: 1-2.
35. Garcia-Fuster MJ, Forner MJ, Flor-Lorente B, et al. Inferior vena cava malformations and deep venous thrombosis. Rev Esp Cardiol 2006; 59: 171-175.
36. Gil RJ, Perez AM, Arias JB, et al. Agenesis of the inferior vena cava associated with lower extremities and pelvic venous thrombosis. J Vasc Surg 2006; 44: 1114-1116.
37. Ilijic M, Krpan M, Banfic L, et al. Postpartal deep-vein thrombosis revealing agenesis of the inferior vena cava. Eur J Obstr Gynecol Reprod Biol 2007; 131: 235-236.
38. Dudeck O, Zeile M, Poellinger A, et al. Epidural venous enlargements presenting with intractable lower back pain and sciatica in a patient with absece of the infrarenal inferior vena cava and bilateral deep venous thrombosis. Spine 2007; 32: 688-691.
39. Evanchuk DM, Von Gehr A, Zehnder JL. Superficial venous thrombosis associated with congenital absence of the inferior vena cava and previous episode of deep venous thrombosis. Am J Hematol 2008; 83: 250-252.
40. Vucicevic Z, Degoricija V, Alfirevic Z, et al. Inferior vena cava agenesia and a massive bilateral iliofemoral venous thrombosis. Angiology 2008; 59: 510-513.
41. Iqbal J, Nagaraju E. Congenital absence of inferior vena cava and thrombosis: a case report. J Med Case Rep 2008; 12: 46.
42. Clayburgh DR, Yoon JD, Cipriani NA, et al. Collateral damage. N Engl J Med 2008; 359: 1048-1054.
43. Tofigh AM, Coscas R, Koskas F, et al. Surgical management of deep venous insufficiency caused by congenital absence of the infrarenal inferior vena cava. Vasc Endovascular Surg 2008; 42: 58-61.
44. Waseem M, Aslam M, Kumar K, et al. An adolescent with tight pain. Pediatr Emerg Care 2008; 24: 768-770.
45. Sanchez Fernandez GL, Reiss UM, de Alarcon PA. Risk of thrombosis with anomalies of the inferior vena cava and factor V Leiden. Pediatr Blood Cancer 2008; 50: 731.
46. Suh HJ, Kim WT, Kim MY, et al. Combined anomaly of the right hepatic lobe agenesis and absence of the inferior vena cava: a case report. Korean J Radiol 2008; 9: 61-64.
47. Kara M, Özcakar L, Eken G, et al. Deep venous thrombosis and inferior vena cava agenesis causing double crush sciatic neuropathy in Behcet’s disease. Joint Bone Spine 2008; 75: 734-736.
48. Kelly J, Sheppard DG, McMullin L. Abdominal pain: a presentation of a vena cava aplasia complicated by thrombosis. Emerg Radiol 2009; 16: 323-326.
49. Vasco PG, Lopez AR, Pineiro ML, et al. Deep venous thrombosis caused by congenital inferior vena cava agenesis and heterozygous factor V Leiden mutation – a case report. Int J Angiol 2009; 18: 147-149.
50. Kondo Y, Koizumi J, Nishibe M, et al. Deep venous thrombosis caused by congenital absence of the inferior vena cava: report of a case. Surg Today 2009; 39: 231-234.
51. Rose SS, Ali Y, Kumar A, et al. Deep venous thrombosis caused by inferior vena cava atresia and hereditary thrombophilia. Am J Med Sci 2009; 337: 67-70.
52. Schierl M, Fiegl N, Gunek-Zalodek S, et al. Becken-Beinvenenthrombose bei kongenitaler Aplasie der Vena cava inferior. Z Gefässmed 2009; 6: 18-20.
53. Guanella R, Glauser F, Bounameaux H, et al. Inferior vena cava agenesis: association with bilateral lower-limb deep vein thrombosis in young males. Thromb Haemost 2009; 102: 795-798.
54. Nichols JL, Gonzalez SC, Bellino PJ, et al. Venous thrombosis and congenital absence of inferior vena cava in a patient with menorrhagia and pelvic pain. J Pediatr Adolesc Gynecol 2010; 23: 17-21.
55. Ismail EA, Azab AF, Jayappa S, et al. [Congenital absence of the infrahepatic segment of the inferior vena cava with deep venous thrombosis in an 8.5-year-old boy.](http://www.ncbi.nlm.nih.gov/pubmed/20500459) Pediatr Int 2010; 52: 117-120.
56. Singh K, Poliquin J, Syversten G, et al. A rare cause of venous thrombosis: congenital absence (agenesis) of the inferior vena cava. Int J Angiol 2010; 19: 110-112.
57. Lambert M, Marboef P, Midulla M, et al. Inferior vena cava agenesis and deep vein thrombosis: 10 patients and review of the literature. Vasc Med 2010; 15: 451-459.
58. O’Conner DB, O’Brien N, Khani T, et al. Superficial and deep vein thrombosis associated with congenitl absence of the infrahepatic inferior vena cava in a young male patient. Ann Vasc Surg 2011; 25: 697e1-4.
59. La Spada M, Stilo F, Carella G, et al. Thrombectomy and surgical reconstruction for extensive iliocaval thrombosis in a patient with agenesis of the retrohepatic vena cava and atresia of the left renal vein. Ann Vasc Surg 2011; 25: 839e1-4.
60. Nseir W, Mahamid M, Abu-Rahmeh Z, et al. Recurrent deep venous thrombosis in a patient with agenesis of inferior vena cava. Int J Gen Med 2011; 4: 457-459.
61. Garg K, Cayne N, Jacobowitz G. Mechanical and pharmacologic catheter-directed thrombolysis treatment of severe, symptomatic, bilateral deep vein thrombosis with congenital absence of the inferior vena cava. J Vasc Surg 2011; 53: 1707-1710.
62. Staubach S, Hug M, Mudra H. Painful congestion of the lower limbs and recurrent fever. Blood Coagul Fibrinolysis 2011; 22: 752-755.
63. Sarlon G, Bartoli MA, Muller C, et al. Congenital anomalies of inferior vena cava in young patients with iliac deep venous thrombosis. Ann Vasc Surg 2011; 25: 265e5-8 .
64. Ganguli S, Kalva S, Oklu R, et al. Efficiacy of lower-extremity venous thrombolysis in the setting of congenital absence or atresia of the inferior vena cava. Cardiovasc Intervent Radiol 2012; 35: 1053-1058.
65. Zinser MJ, Hanto DW. Liver transplantation in a patient with developmental interruption of the inferior vena cava with azygos substitution. Transplant Proc 2012; 44: 1460-1463.
66. Lavens M, Moors B, Thomis S. Deep vein thrombosis in a young female with hypoplastic inferior vena cava. Ann Vasc Surg 2013; 28:1036.e5-7.
67. Skeik N, Wickstrom KK, Schumacher CW, et al. Infrahepatic inferior vena cava agenesis with bilateral renal vein thrombosis. Ann Vasc Surg 2013; 27: 973e19-23.
68. Yugueros X, Alvarez B, Fernandez E, et al. Compressive symptoms due to thrombosed or hypertrophic collateral circulation in infrarenal inferior vena cava agenesis. Ann Vasc Surg 2013; 27: 238e9-13.
